# New insight into the SSC8 genetic determination of fatty acid composition in pigs

**DOI:** 10.1186/1297-9686-46-28

**Published:** 2014-04-23

**Authors:** Manuel Revilla, Yuliaxis Ramayo-Caldas, Anna Castelló, Jordi Corominas, Anna Puig-Oliveras, Noelia Ibáñez-Escriche, María Muñoz, Maria Ballester, Josep M Folch

**Affiliations:** 1 Centre de Recerca en Agrigenòmica (CRAG), Consorci CSIC-IRTA-UAB-UB, Campus UAB, Bellaterra 08193, Spain; 2 Departament de Ciència Animal i dels Aliments, Facultat de Veterinària, Universitat Autònoma de Barcelona, Bellaterra 08193, Spain; 3 Genètica i Millora Animal, IRTA, Av Rovira Roure 191, Lleida 25198, Spain; 4 Departamento Mejora Genética Animal, SGIT-INIA, Ctra. Coruña Km 7.5, Madrid 28040, Spain

## Abstract

**Background:**

Fat content and fatty acid composition in swine are becoming increasingly studied because of their effect on sensory and nutritional quality of meat. A QTL (quantitative trait locus) for fatty acid composition in backfat was previously detected on porcine chromosome 8 (SSC8) in an Iberian x Landrace F_2_ intercross. More recently, a genome-wide association study detected the same genomic region for muscle fatty acid composition in an Iberian x Landrace backcross population. *ELOVL6*, a strong positional candidate gene for this QTL, contains a polymorphism in its promoter region (*ELOVL6:c.-533C < T*), which is associated with percentage of palmitic and palmitoleic acids in muscle and adipose tissues. Here, a combination of single-marker association and the haplotype-based approach was used to analyze backfat fatty acid composition in 470 animals of an Iberian x Landrace F_2_ intercross genotyped with 144 SNPs (single nucleotide polymorphisms) distributed along SSC8.

**Results:**

Two trait-associated SNP regions were identified at 93 Mb and 119 Mb on SSC8. The strongest statistical signals of both regions were observed for palmitoleic acid (C16:1(n-7)) content and C18:0/C16:0 and C18:1(n-7)/C16:1(n-7) elongation ratios. *MAML3* and *SETD7* are positional candidate genes in the 93 Mb region and two novel microsatellites in *MAML3* and nine SNPs in *SETD7* were identified. No significant association for the *MAML3* microsatellite genotypes was detected. The *SETD7:c.700G > T* SNP, although statistically significant, was not the strongest signal in this region. In addition, the expression of *MAML3* and *SETD7* in liver and adipose tissue varied among animals, but no association was detected with the polymorphisms in these genes. In the 119 Mb region, the *ELOVL6:c.-533C > T* polymorphism showed a strong association with percentage of palmitic and palmitoleic fatty acids and elongation ratios in backfat.

**Conclusions:**

Our results suggest that the polymorphisms studied in *MAML3* and *SETD7* are not the causal mutations for the QTL in the 93 Mb region. However, the results for *ELOVL6* support the hypothesis that the *ELOVL6:c.-533C > T* polymorphism has a pleiotropic effect on backfat and intramuscular fatty acid composition and that it has a role in the determination of the QTL in the 119 Mb region.

## Background

One of the main sources of human-consumed meat is pork, which represents more than 40% of the meat produced worldwide [1]. The success of pig production is strongly related to improvements in growth and carcass yield. Meat-quality traits are essential for the processing industry and end-consumer acceptance [2], and, as a result, these qualitative traits have been the subject of many studies in breeding programs. Fat content and fatty acid (FA) composition in swine are becoming increasingly studied because of their effect on sensory and nutritional quality of meat. They determine important sensory and technological aspects of pork and meat products because of their influence on the melting point and oxidative status of porcine tissues [3]. In addition, the amount and type of fat in the diet have a major impact on human health. The high consumption of saturated fatty acids (SFA) raises plasma LDL-cholesterol, which is a major risk factor for arteriosclerosis and coronary heart disease (CHD) [4-6]. However, recent studies suggest that individual SFA have different physiological effects. Indeed, lauric acid (12:0), myristic acid (14:0) and palmitic acid (16:0) raise LDL and HDL cholesterol plasma levels, whereas stearic acid (C18:0) is considered neutral [7,8], although some epidemiologic evidence suggests that stearic acid (C18:0) is associated with CHD [9]. In contrast, cis-monounsaturated fatty acids (MUFA) and polyunsaturated fatty acids (PUFA) are beneficial for human health. PUFA have been shown to protect against CHD [10], whereas MUFA are also considered to have a hypocholesterolemic effect [11] and, in addition, to have a beneficial effect on insulin sensitivity [12].

A few QTL (quantitative trait loci) for FA composition have been reported on porcine chromosome 8 (SSC8) in F_2_ Duroc x Large White [13], F_2_ White Duroc x Erhualian [14] and Duroc [15] populations. Using an Iberian x Landrace F_2_ intercross (IBMAP) [16], a QTL was identified on SSC8 for percentages of palmitic (16:0) and palmitoleic (C16:1(n-7)) FA and for average length of FA in backfat (BF). Recently, a genome-wide association study (GWAS) conducted in a backcross population (BC1_LD; 25% Iberian and 75% Landrace) led to the identification of five genomic regions on SSC8 associated with intramuscular fat in *longissimus dorsi* (IMF) FA composition [17]. In addition, a study that combined a linkage QTL scan and a GWAS on the same backcross revealed significant pleiotropic regions with effects on palmitic (C16:0) and palmitoleic (C16:1(n-7)) FA in both IMF and BF tissues [18].

The main goals of this work were: (1) to study the QTL architecture for FA composition on SSC8 in the F_2_ generation of the IBMAP cross using a panel of 144 informative SNPs, and (2) to analyze additional positional candidate genes.

## Methods

### Animal samples

Animals used in this study belong to the IBMAP experimental population [19]. Two Iberian (Guadyerbas line) boars were crossed with 30 Landrace sows to generate the F_1_ generation. Six F_1_ boars were coupled with 67 F_1_ sows to obtain 470 F_2_ animals. In addition, gene-expression analyses were carried out on 56 females from a backcross (BC1_LD) generated by crossing five F_1_ (Iberian x Landrace) boars with 23 Landrace sows. All animals were maintained under intensive conditions and feeding was *ad libitum* with a cereal-based commercial diet. The experiments were performed in Europe following national and institutional guidelines for the ethical use and treatment of animals in experiments. In addition, the protocol was approved by the Ethical Committee of the Institution (IRTA Institut de Recerca i Tecnologia Agroalimentàries). F_2_ animals were slaughtered at an average age of 175.5 ± 0.3 days. However, tissues for RNA extraction were not isolated from animals of the F_2_ generation. Backcross animals were slaughtered at an average age of 179.8 ± 2.6 days, and samples of liver and adipose tissue were collected, snap-frozen in liquid nitrogen and stored at -80°C until analysis.

Genomic DNA was extracted from blood samples of all animals by the phenol-chloroform method, as described elsewhere.

### Traits analyzed

The composition of 10 FA in IMF and BF (taken between the third and the fourth ribs) tissues was determined by gas chromatography as described in [16,17,19]. Subsequently, the percentage of each FA, relative to the total FA, was calculated as well as the global percentages of SFA, MUFA, PUFA and related indices, including desaturation and elongation indices.

### Genotyping and quality control

A total of 470 animals were genotyped for 144 SNPs located on SSC8; these include a selection of 142 informative SNPs derived from the Porcine SNP60K BeadChip [20] and two SNPs that corresponded to the previously detected polymorphisms in the *FABP2*[21] and *MTTP*[22] genes. These SNPs [See Additional file 1: Table S1] were included in a custom-generated panel, genotyped using a Veracode Golden Gate Genotyping Kit (Illumina Inc.) and analyzed with a Bead Xpress Reader (Illumina Inc.). SNP positions were based on the whole-genome sequence assembly 10.2 build of *Sus scrofa* (http://www.animalgenome.org/repository/pig/). All genotypes were assigned using the GenomeStudio software (Illumina Inc.). Markers that had a minor allele frequency (MAF) lower than 5% and missing genotypes that had a frequency greater than 5% were removed using PLINK [23] software. In total, 133 SNPs (92%) passed this quality-threshold filter and were used in the subsequent analysis. Genotypes of all the parents were obtained with the 60 K SNP chip (Illumina) [17] or by pyrosequencing [21,22].

SNPs *SETD7:c.-1034T > G*, *SETD7:c.700G > T* and *ELOVL6:c.-533C > T* were genotyped using the KASP SNP genotyping system platform (http://www.lgcgenomics.com/genotyping/). Besides these, two new microsatellites in the *MAML3* gene were genotyped by PCR amplification and capillary electrophoresis and fluorescent detection using an ABI Prism 3730 DNA Analyzer (Applied Biosystems).

Fifty-six animals of the BC1_LD were genotyped for SNPs *SETD7:c.-1034T > G* and *SETD7:c.700G > T* and the two *MAML3* microsatellites for gene-expression studies. In addition, a subset of 168 F_2_ animals were genotyped for SNPs *ELOVL6:c.-533C > T*, *SETD7:c.700G > T* and the two *MAML3* microsatellites for association studies. All parents and grandparents of these animals were also genotyped in the same way.

### Association analysis

Association analysis was performed for FA composition and indices of FA metabolism in 470 F_2_ animals. A mixed model that accounts for additive effects was performed using Qxpak 5.0 [24]:

$$y_{\mathrm{ijlkm}}={\mathrm{Sex}}_{i}+{\mathrm{Batch}}_{j}+\beta c_{l}+\lambda_{l}a_{k}+u_{l}+e_{\mathrm{ijlkm}},$$

where y_ijlkm_ is the l^th^ individual record, sex (two levels) and batch (five levels) are fixed effects, β is a covariate coefficient with c being carcass weight, λ_l_ is a -1, 0, +1 indicator variable depending on the l^th^ individual genotype for the k^th^ SNP, a_k_ represents the additive effect associated with SNP, u_l_ represents the infinitesimal genetic effect treated as random and distributed as N(0, **A**σ_u_) where **A** is a numerator of the kinship matrix and e_ijlkm_ is the residual. A similar model that fitted different QTL effects was used to test the hypothesis of the presence of two QTL located in the studied regions with effects a_1_ and a_2_ on the same FA:

$$\begin{array}{ll} y_{\mathrm{ijlkm}} & ={\mathrm{Sex}}_{i}+{\mathrm{Batch}}_{j}+\beta c_{l}+\lambda_{l}a_{1k}+\lambda_{l}a_{2k}+u_{l}\\ & \;+e_{\mathrm{ijlkm}}, \end{array}$$

The R package q-value [25] was used to calculate the false-discovery rate (FDR), and the cut-off of the significant association at the whole-genome level was set at the q-value ≤ 0.05. Version 2.15.2 of R [26] was used to calculate the descriptive statistics for the 10 analyzed traits and their related indices.

For linkage and linkage disequilibrium (LDLA) analysis, haplotypes were reconstructed using DualPHASE software [27], which exploits population (linkage disequilibrium) and family information (Mendelian segregation and linkage) in a Hidden Markov Model setting. Then, QTL fine-mapping was performed for the most significant traits C16:1(n-7), C18:0/C16:0, C16:1(n-7)/C18:1(n-7), and the FA average chain-length (ACL) by applying the mixed model:

$$y=\mathrm{Xb}+Z_{h}h+Z_{u}u+e,$$

in which **b** is a vector of fixed effects (sex and batch), **h** is the vector of random QTL effects corresponding to the K cluster defined by the Hidden State, **u** is the vector of random individual polygenic effects and **e** is the vector of individual error.

### Amplification and sequencing of the pig *MAML3* and *SETD7* genes

Genomic DNA samples from 10 individuals of the BC1_LD and two Iberian boars were used to amplify and sequence the proximal promoter and exon 1 of the *MAML3* and *SETD7* genes.

A 931-bp region of the *MAML3* gene was amplified and sequenced in two overlapping fragments of 517 bp and 663 bp. Primers [See Additional file 2: Table S2] were designed based on a SSC8 sequence of a *Sus scrofa* mixed breed [ENSSSCG00000009060] available from the *Sscrofa10.2* database and conserved with the human *MAML3* gene [ENSG00000196782].

For the *SETD7* gene, two overlapping fragments of 473 bp and 478 bp were amplified and sequenced. Primers [See Additional file 2: Table S2] were designed based on a SSC8 sequence of a *Sus scrofa* mixed breed [ENSSSCG00000030396] available from the *Sscrofa10.2* database and conserved with the human *SETD7* gene [ENSG00000145391].

All primers were designed using the PRIMER3 software [28] and were validated using the PrimerExpress 2.0 software (Applied Biosystems).

PCR (polymerase chain reactions) were carried out in a total volume of 25 μL containing 0.6 units of AmpliTaq Gold (Applied Biosystems), 1.5 to 2.5 mM MgCl_2_ depending on the primers [See Additional file 2: Table S2], 0.2 mM of each dNTP, 0.5 μM of each primer and 20 ng of genomic DNA. The temperature profile was 94°C for 10 min and 35 cycles at 94°C for 1 min, 58°C to 62°C depending on the primers [See Additional file 2: Table S2] for 1 min and 72°C for 1.5 min, including a final step of 7 min at 72°C. Gradient parameters were determined based on size and GC content of the amplicon. The samples were then analyzed on 1.5% agarose gels. Purification was performed using an Exonuclease I and FastAP™ Thermosensitive Alkaline Phosphatase [29]. For the sequencing reaction, we used the Big Dye Terminator v.3.1 Cycle Sequencing Kit and an ABI Prism 3730 DNA analyzer was employed (Applied Biosystems). Polymorphisms were checked through the Seq scape v2.1.1 program (Applied Biosystems).

### Detection of microsatellite polymorphisms

Based on the sequencing results of the promoter region and exon 1 of the *MAML3* gene, two new microsatellites were identified. Both microsatellites were independently amplified using fluorescent primers [See Additional file 2: Table S2]. PCR were performed in a 25-μL reaction mix containing 20 ng of genomic DNA, 0.2 mM of each dNTP, 2.5 mM MgCl_2_, 0.5 μM of each PCR primer and 0.6 units of AmpliTaq Gold (Applied Biosystems). PCR were run as follows: 94°C for 10 min, 35 cycles of 94°C for 1 min, 58°C for 1 min, 72°C for 1.5 min and a final extension step at 72°C for 7 min. The two amplicons were mixed at a ratio of 1:3 (HEX:FAM) and analyzed using capillary electrophoresis on an ABI Prism 3730 DNA analyzer (Applied Biosystems) and the ROX-500 GeneScan Size Standard. The peak height of each product was determined using Peak Scanner 2 software (Applied Biosystems).

### RNA isolation and cDNA synthesis

Total RNA was extracted from liver and BF tissues using the RiboPure kit (Ambion), according to the manufacturer’s recommendations. RNA was then quantified using a NanoDrop ND-1000 spectrophotometer (NanoDrop products) and RNA integrity was assessed with an Agilent Bioanalyzer-2100 (Agilent Technologies). One μg of total RNA of each sample was reverse-transcribed using the High-Capacity cDNA Reverse Transcription kit (Applied Biosystems) in a reaction volume of 20 μL.

### Gene-expression quantification

Fifty-six females of the BC1_LD were used to quantify gene expression. The expression of *MAML3* and *SETD7* was analyzed using the 48.48 microfluidic dynamic array IFC chip (Fluidigm) according to the manufacturer’s instructions. Briefly, 2 μL of 1:5 diluted cDNA was pre-amplified using 2X Taqman PreAmp Master Mix (Applied Biosystems) and 50 nM of each primer pair in 5-μL reaction volume. The cycling program consisted of an initial step of 10 min at 95°C followed by 16 cycles of 15 s at 95°C and 4 min at 60°C. At the end of this pre-amplification step, the reaction products were diluted 1:5 (diluted pre-amplification samples). RT-qPCR on the dynamic array chips was conducted on the BioMarkTM system (Fluidigm). A 5-μL pre-mix sample containing 2.5 μL of SsoFast EvaGreen Supermix with Low ROX (Bio-Rad), 0.25 μL of DNA Binding Dye Sample Loading Reagent (Fluidigm) and 2.25 μL of diluted pre-amplification samples (1:16 or 1:64 from the diluted pre-amplification samples from liver and BF samples, respectively), as well as a 5-μL assay mix containing 2.5 μL of Assay Loading Reagent (Fluidigm), 2.25 μL of DNA Suspension Buffer (Teknova) and 0.25 μL of 100 μM primer pairs (500 nM in the final reaction) were mixed inside the chip using the IFC controller MX (Fluidigm). The cycling program consisted of an initial step of 60 s at 95°C followed by 30 cycles of 5 s at 96°C and 20 s at 60°C. A dissociation curve was also drawn for each primer pair.

Data were collected using the Fluidigm Real-Time PCR analysis software 3.0.2 (Fluidigm) and analyzed with the DAG expression software 1.0.4.11 [30] using standard curves for relative quantification. Relative standard-curves with a four-fold dilutions series (1/4, 1/16, 1/64, 1/256, 1/1024) of a pool of 10 cDNA samples were constructed for each gene to extrapolate the value of the quantities of each studied sample. Of the four endogenous genes tested (*ACTB*, *B2M*, *HPRT1*, *TBP*), *ACTB* and *TBP* had the most stable expression [31] in both tissues. The normalized quantity values of each sample and assay were used to compare our data.

PCR primer sequences [See Additional file 2: Table S2] were designed using PrimerExpress 2.0 software (Applied Biosystems).

Mean values between genotypes were compared using a linear model implemented in R, which performs a single stratum analysis of variance considering sex and batch as fixed effects. Differences were considered statistically significant at a p-value of 0.05.

## Results and discussion

### Association studies and combined linkage disequilibrium and linkage analyses

A custom panel of 144 SNPs located on SSC8 was used to genotype 470 F_2_ animals. Association analyses for the BF FA composition in the C14:0 to C22:0 ranges were performed with genotypes from a subset of 133 SNPs (call rate > 0.99). Statistically significant associations were found (Table 1) for the SFA myristic (C14:0), palmitic (C16:0) and stearic acids (C18:0). Among MUFA, palmitoleic acid (C16:1(n-7)) and oleic acid (C18:1(n-9)) were associated, whereas for PUFA only eicosadienoic acid (C20:2(n-6)) was significant. Similarly, the ACL metabolic ratio showed a significant association. A strong association signal was found for the C16:1(n-7)/C16:0 desaturation ratio and two elongation ratios: C18:0/C16:0 and C18:1(n-7)/C16:1(n-7).

**Table 1:** **Significant SNPs affecting BF FA composition (FDR = 0.05) in an association study with 470 animals of the F**_**2 **_**generation**

**Trait**	**Chromosomal region (Mb)**	**SNP**	**LR**	***P*-value**	***a *** **(SE)**
**C14:0**	93.79	ALGA0048597	17.2127	3.34E-05	0.042 (0.019)
	117.55	ALGA0049135	18.7843	1.46E-05	0.055 (0.016)
**C16:0**	93.72	ALGA0048594	32.4610	1.22E-08	0.573 (0.881)
	117.66	ALGA0049139	48.1404	3.97E-12	0.599 (0.871)
**C18:0**	91.56	H3GA0025111	11.7215	6.18E-04	-0.254 (0.630)
	119.85^1^	INRA0030422	20.6040	5.65E-06	-0.366 (0.616)
**C16:1(n-7)**	91.56	H3GA0025111	42.9598	5.59E-11	0.163 (0.082)
	119.85^1^	INRA0030422	71.7870	1.11E-18	0.223 (0.082)
**C18:1(n-9)**	93.66	ALGA0048589	22.4009	2.21E-06	-0.651 (1.589)
	117.66	ALGA0049139	33.1059	8.73E-09	-0.672 (1.571)
**C20:2(n-6)**	94.73	MARC0097057	23.1170	1.52E-06	-0.032 (0.017)
	117.55	ALGA0049135	23.9259	1.00E-06	-0.039 (0.017)
**ACL**	93.72	ALGA0048594	46.5350	9.00E-12	-0.020 (0.001)
	117.66	ALGA0049139	71.4236	1.11E-16	-0.021 (0.001)
**C16:1(n-7)/C16:0**	91.56	H3GA0025111	22.7521	1.84E-06	0.006 (0.000)
	119.85^1^	INRA0030422	37.4524	9.37E-10	0.008 (0.000)
**C18:0/C16:0**	91.56	H3GA0025111	47.8703	4.55E-12	-0.023 (0.002)
	119.85^1^	INRA0030422	76.1635	1.11E-16	-0.032 (0.002)
**C18:1(n-7)/C16:1(n-7)**	93.72	ALGA0048594	32.8062	1.02E-08	-0.076 (0.016)
	119.85^1^	INRA0030422	61.0692	5.55E-15	-0.089 (0.015)
**C20:2(n-6)/C18:2(n-6)**	120.99	ALGA0049254	13.8456	1.98E-04	-0.003 (0.000)

LR = Likehood ratio test values; *a* (SE): additive effect (standard error); ^1^SNPs SIRI0000509 (119.73 Mb) and H3GA0025321 (119.89 Mb) showed the same *P*-value.

Two regions that contain trait-associated SNPs (TAS) were clearly visualized in the association plots at around 93 Mb and 119 Mb for all of the above-mentioned FA and indices with the exception of the C20:2(n-6)/C18:2(n-6) elongation ratio, which showed only one significant TAS region at 120.99 Mb (Table 1). For all significant traits, the 119 Mb TAS region showed a stronger signal than the 93 Mb region. The strongest effects of both TAS regions were found for palmitoleic acid (C16:1(n-7)) content and C18:0/C16:0 and C18:1(n-7)/C16:1(n-7) elongation ratios.

**Figure 1:**
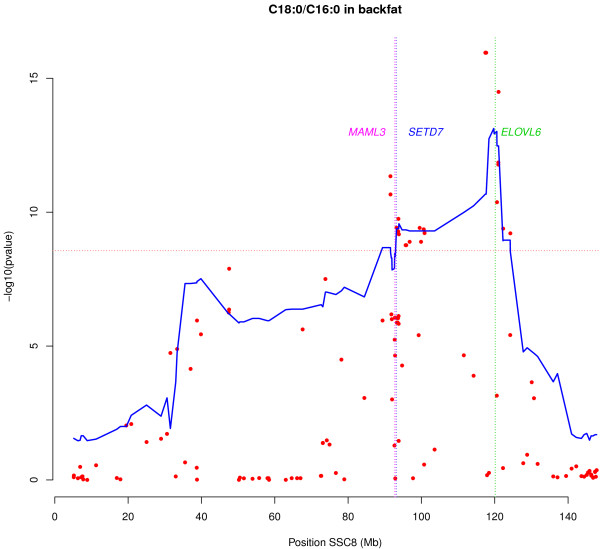
**Association study and LDLA of the C18:0/C16:0 elongation ratio in BF.** Plot of association study (red points) and LDLA patterns (blue line) for the stearic/palmitic ratio; the X-axis represents positions in Mb on SSC8, and the Y-axis shows the –log10 (p-value); vertical, the pink line represents the position of the *MAML3* gene, the blue line represents the position of the *SETD7* gene and the green line represents the position of the *ELOVL6* gene on SSC8; horizontal, dashed lines mark the association study significance level (FDR-based q-value ≤ 0.05); positions in Mb are relative to the *Sscrofa10.2* assembly of the pig genome.

A combination of linkage disequilibrium and linkage analysis (LDLA) was then performed for the most significantly associated traits [See Additional file 2: Table S3]. With this haplotype-based approach, it is possible to simultaneously exploit linkage analysis and linkage disequilibrium. Several studies have shown the usefulness of this strategy for fine-mapping and QTL interval reduction [27,32]. The LDLA study identified the two TAS regions by association analysis, with the 119 Mb region showing the strongest statistical signal for all analyzed traits. Figure 1 shows the two genomic regions identified for the C18:0/C16:0 elongation ratio. Plots of the other three traits analyzed are shown in Additional file 3: Figure S1.

In order to determine whether one or two QTL were segregating on SSC8 for the BF FA and their indices, models fitting one QTL against a model considering two different QTL were tested. Results of the LR test indicated that the model with two QTL was the most likely for the 10 traits analyzed [See Additional file 2: Table S4].

Previously, a QTL scan for BF FA composition was performed with 369 animals from the same F_2_ generation [16], but only six microsatellite markers were genotyped. A clear effect of SSC8 markers was observed only for percentages of palmitic (C16:0) and palmitoleic (C16:1(n-7)) FA and ACL. A suggestive effect on percentage of oleic acid (C18:1(n-9)) was also observed. However, the confidence interval for this QTL was greater than 30 cM. Two other studies of our group analyzed positional candidate genes for this QTL, i.e. *FABP2*[21] and *MTTP*[22], but the localization of the QTL was not refined. In addition, QTL for IMF palmitic (C16:0) FA composition have been reported in a Duroc x Large White F_2_ cross [13] and for stearic (C18:0) FA in a White Duroc x Erhualian F_2_ cross [14].

A GWAS for IMF FA composition [17] with genotypes from the 60 K SNP chip (Illumina) was carried out using 144 animals from a related backcross population (BC1_LD). The strongest signals on SSC8 were observed for the palmitoleic (C16:1(n-7)) FA content and the C18:1(n-7)/C16:1(n-7) ratio for SNPs ALGA0048684 and SIRI0000509, which in the *Sscrofa10.2* assembly are located at 99.2 Mb and 119.7 Mb, respectively. Furthermore, two significant pleiotropic regions (at 93.3 Mb - 99.5 Mb and 110.9 Mb - 126.9 Mb) with effects on palmitoleic (C16:1(n-7)) FA in both IMF and BF tissues have been identified in the same backcross [18]. For palmitic (C16:0) FA, a large (83.8 Mb - 130.6 Mb) chromosomal interval was significant for both BF and IMF [18].

Here, two QTL at approximately 93 Mb and 119 Mb were detected and affected the BF composition of the six FA and the four indices mentioned above in the 470 F_2_ animals. The palmitoleic (C16:1(n-7)) FA QTL on SSC8 have been shown to be segregating in different crosses of the IBMAP population, and both QTL have a pleiotropic effect on BF and IMF FA deposits.

### Gene annotation and identification of polymorphisms in positional candidate genes

Gene annotation of the two TAS genomic regions allowed us to identify genes related to FA metabolism. In the first region, the genes *mastermind-like 3* (*MAML3*) (at position 92.67 Mb) and *SET domain containing lysine methyltransferase 7* (*SETD7*) (at position 93.13 Mb) were found. Both genes have recently been reported in a predicted co-association gene network for intramuscular FA composition in pigs (Ramayo *et al*., 2013; unpublished observations).

*MAML3* is a member of the *Mam* gene family, which plays an essential role in the stabilization of Notch transcriptional activation complexes [33]. This Notch signaling pathway mediates short-range communication between cells, and it has recently been associated with the regulation of lipogenesis and gluconeogenesis in liver [34]. A 931-bp fragment of the pig *MAML3* gene that covers part of the promoter region and part of exon 1, was amplified from genomic DNA and sequenced. Two novel microsatellites were found: *MAML3_MS1,* a (CA)_n_ tandem repeat located in the promoter region and *MAML3_MS2,* a (CGG)_n_ tandem repeat identified in exon 1. The variability of both microsatellites is described in Table 2.

**Table 2:** **Microsatellites identified in the *MAML3 *** **gene**

**SSR locus**	**Repeat**	**5’ fluorescent label**	**Number of alleles**	**Size of alleles**
*MAML3_MS1*	(CA)n	HEX	8	233,239,243,245,247,251,257,259
*MAML3_MS2*	(CGG)n	FAM	2	135,138

The product of the *SETD7* gene is a histone methyltransferase that specifically monomethylates Lys-4 of histone H3 [35] and, thus, it is involved in the epigenetic transcriptional regulation of genes, activating genes such as *collagenase* or *insulin*[36]. To identify polymorphims in the porcine *SETD7* gene, a 839-bp fragment of the *SETD7* promoter and exon 1 was amplified from genomic DNA and sequenced. In addition, the identification of polymorphisms in the entire coding region of the *SETD7* gene was performed using *RNA-Seq* data [37] with the Integrative Genomics Viewer (IGV) software (http://www.broadinstitute.org/igv/). Alignment and analysis of these sequences led to the identification of nine polymorphisms (Table 3). Two of these polymorphisms were used to genotype BC1_LD animals, one located in the promoter region (*SETD7:c.-1034T > G*) and one non-synonymous polymorphism in exon 6 (*SETD7:c.700G > T*), which determines an amino-acid change of valine to leucine. Apart from the fact that these SNPs are located in the *SETD7* gene, they were selected because they showed divergent allelic frequencies between the Iberian and Landrace IBMAP founders i.e. the *SETD7:c.-1034 T* and *SETD7:c.700 T* alleles were fixed in the Iberian boars. Complete linkage disequilibrium between the two SNPs was observed in the genotyped BC1_LD animals and, thus, only *SETD7:c.700G > T* was further genotyped in 168 animals belonging to the F_2_ generation.

**Table 3:** **Polymorphisms identified in the proximal promoter and coding regions of the *SETD7 *** **gene**

**Gene localization**	**Position (bp)**	**Ref**^**4**^	**Pol**^**5**^	**Aminoacid change**
**Promoter**^**1**^	-1300	A	G	
	-1034^3^	T	G	
	-980	C	A	
	-632	T	C	
**Exon 4**^**2**^	462	C	T	
**Exon 6**^**2**^	700^3^	G	T	VAL/LEU
	708	G	A	
**Exon 7**^**2**^	807	C	T	
**Exon 8**^**2**^	960	C	T	

^1^Positions relative to the transcription start-site using, as reference, the GenBank ENSSSCG00000030396 sequence; ^2^referring to the coding region, using RNA-Seq data; ^3^SNPs genotyped; ^4^Ref = nucleotide in the reference sequence; ^5^Pol = polymorphisms found.

In the second region, the *ELOVL6* gene was identified at position 120.12 Mb. The *ELOVL6* gene is a strong positional and functional candidate gene involved in *de novo* lipogenesis and acts on the elongation of SFA and MUFA. A polymorphism in the promoter region of this gene (*ELOVL6:c.-533C > T*) has previously been associated with percentages of palmitic and palmitoleic FA in muscle and backfat in the BC1_LD population [38]. In addition, expression of the *ELOVL6* gene was lower in the backfat of animals with the Iberian allele in comparison to those with the Landrace allele. As expected from the elongation function of this gene, a lower *ELOVL6* expression was associated with a higher percentage of palmitic and palmitoleic FA in muscle and adipose tissue [38].

**Figure 2:**
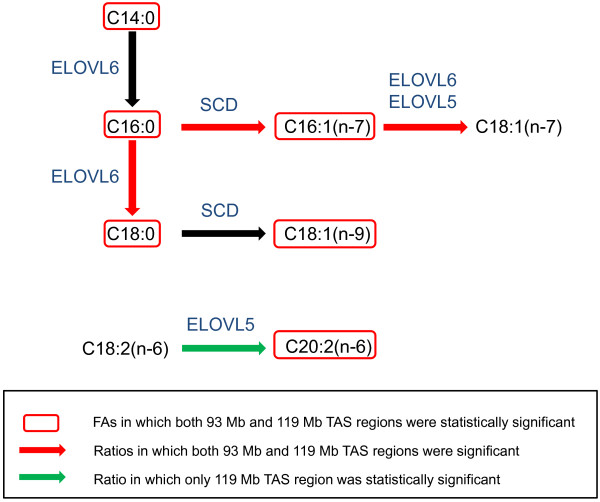
**Schematic representation of the elongation pathway of 16 and 18 carbon FAs.** Statistically significant FA (red square) and ratios of elongation and desaturation (colored arrows) are shown for BF FA composition in the F_2_ generation.

Based on our results, the observed effects on FA composition and indices are concordant with a lower expression of the *ELOVL6* gene in animals with the Iberian allele [38] for both TAS regions (Figure 2). *ELOVL6* elongates palmitic (C16:0) to stearic (C18:0), and palmitoleic (C16:1(n-7)) to vaccenic (C18:1(n-7)) FA. Thus, a lower ELOVL6 activity associated with the Iberian allele will directly decrease these elongation ratios. Moreover, as observed, a lower ELOVL6 activity will result in the accumulation of palmitic (C16:0) and palmitoleic (C16:1(n-7)) FA and a reduction in stearic (C18:0) FA content (Figure 2).

Regarding oleic FA (C18:1(n-9)), the main dietary FA, its content in BF was decreased in animals with the Iberian allele for the TAS regions on SSC8. It must be noted that the opposite effect was observed in the major SSC4 and SSC6 TAS regions for oleic (C18:1(n-9)) IMF content [17].

### Effect of the *SETD7:c.1034T > G* and *SETD7:c.700G > T* SNPs and *MAML3* microsatellites on gene expression

The expression profiles of the pig *MAML3* and *SETD7* genes were studied in liver and BF tissues of 56 BC1_LD females by RT-qPCR. The analysis of gene expression in the F_2_ generation was not possible because tissues for RNA isolation were not available. Differences in the expression of *MAML3* among animals were observed, with coefficients of variation (CV) of 35% and 42% in liver and BF, respectively. The *SETD7* gene expression was less variable, with CV values of 18% and 33% in liver and BF tissue, respectively. However, no significant differences in expression of *SETD7* were detected among animals classified according to the *SETD7* genotypes (either *SETD7:c.-1034T > G* or *SETD7:c.700G > T*) in either tissue. Similarly, no differences in expression of *MAML3* were observed among animals classified according to the *MAML3_MS1* and *MS2* microsatellites. In addition, no significant correlation was found between expression levels of *MAML3* or *SETD7* in the liver and adipose, which suggests that different and tissue-specific mechanisms control the liver and adipose tissue expression of *MAML3* and *SETD7*.

### Association study for BF FA composition with markers located in positional candidate genes

Two microsatellites in the *MAML3* gene (*MAML3_MS1* and *MAML3_MS2*), one SNP in the *SETD7* gene (*SETD7:c.700G > T*), and one SNP in the *ELOVL6* gene (*ELOVL6:c.-533C > T*) were used to genotype 168 animals of the F_2_ generation. An association analysis with these markers and the SSC8 genotypes from the 133 SNPs of our custom porcine SNP panel was performed.

For the first region (93 Mb), polymorphisms in the *SETD7* and *MAML3* genes were studied. For *SETD7*, the *SETD7:c.700G > T* polymorphism did not show the most significant association (Table 4). In addition, *MAML3* gene microsatellites showed no significant associations for any of the traits studied. However, the SNPs showing the strongest signals (Table 4) were located within a 2 Mb interval of the *SETD7* and *MAML3* genes. These results suggest that other non-genotyped polymorphisms may cause the observed effects on FA composition in the 93-Mb region.

**Table 4:** **Significant SNPs affecting BF FA composition (FDR = 0.05) in 168 F**_**2 **_**animals**

**Trait**	**Chromosomal region (Mb)**	**SNP**	**LR**	***P*-value**	***a *** **(SE)**
**C16:0**	93.29	MARC0024098	14.3307	1.53E-04	0.645 (0.828)
	120.01	*ELOVL6:c.533C > T*	16.9446	3.85E-05	0.652 (0.818)
**C16:1(n-7)**	93.62	MARC0005229	23.5897	1.19E-06	0.181 (0.049)
	120.01	*ELOVL6:c.533C > T*	33.1038	8.74E-09	0.221 (0.045)
**C18:1(n-9)**	93.29	MARC0024098	23.7323	1.11E-06	-0.838 (0.810)
	117.44	ASGA0039595	24.0369	9.45E-07	-0.827 (0.809)
**C18:1(n-7)**	142.23	ALGA0106925	13.3222	2.62E-04	0.174 (0.049)
**ACL**	93.29	MARC0024098	19.4268	1.05E-05	-0.021 (0.001)
	120.01	*ELOVL6:c.533C > T*	24.7173	6.64E-07	-0.022 (0.001)
**MUFA**	117.44	ASGA0039595	13.8134	2.02E-04	-0.724 (1.108)
**C16:1(n-7)/C16:0**	93.62	MARC0005229	16.2984	5.41E-05	0.007 (0.000)
	127.78	MARC0087394	20.5866	5.70E-06	0.007 (0.000)
**C18:0/C16:0**	93.29	MARC0024098	17.2826	3.22E-05	-0.027 (0.001)
	120.01	*ELOVL6:c.533C > T*	28.3700	1.00E-07	-0.032 (0.001)
**C18:1(n-7)/C16:1(n-7)**	93.77	MARC0020530	26.6172	2.48E-07	-0.092 (0.009)
	120.01	*ELOVL6:c.533C > T*	36.7487	1.34E-09	-0.101 (0.008)
**C20:2(n-6)/C18:2(n-6)**	91.93	ALGA0048544	17.4256	2.99E-05	0.009 (0.000)
	93.62	MARC0005229	15.8393	6.90E-05	-0.004 (0.000)

LR = Likehood ratio test values; *a* (SE) = additive effect (standard error).

For the second region (119 Mb), a polymorphism in the *ELOVL6* gene was studied. The *ELOVL6:c.-533C > T* polymorphism showed the highest association with percentage of palmitic and palmitoleic FA, ACL, and C18:0/C16:0 and C18:1(n-7)/C16:1(n-7) ratios (Table 4). Hence, these results are consistent with those found in the IMF FA composition of the BC1_LD generation [38]. The clear association of the *ELOVL6:c.-533C > T* polymorphism with percentage of FA in IMF and BF indicates a pleiotropic effect of this gene in both tissues.

Analysis of the additive value of SNPs *SETD7:c.700G > T* and *ELOVL6:c.-533C > T* showed a higher contribution of *ELOVL6:c.-533C > T* SNP for all studied FA and indices. Furthermore, the additive value of the two SNPs [See Additional file 2: Table S5] showed an effect in the same direction. For instance, the Iberian alleles of both QTL increased palmitic and palmitoleic FA content and reduced the elongation ratios. These results are in accordance with the reported Iberian-Landrace breed differences in BF FA composition [39].

## Conclusions

In summary, two TAS regions at 93 Mb and 119 Mb on SSC8 affect BF FA composition. Both regions showed a strong effect on palmitoleic acid content and C18:0/C16:0 and C18:1(n-7)/C16:1(n-7) elongation ratios. The *MAML3* and *SETD7* genes were analyzed as positional candidate genes of the 93-Mb TAS region. Two novel microsatellites were identified in the *MAML3* gene, and nine SNPs in the *SETD7* gene. However, the association analysis did not reveal any significant association between the *MAML3* microsatellite genotypes and the traits studied, and the *SETD7:c.700G > T* SNP did have not the strongest signal in the 93 Mb region. Although the expression of *MAML3* and *SETD7* genes in liver and adipose tissue varied among animals, it was not associated with any of the genotyped polymorphisms in these genes. These results suggest that the polymorphisms studied in *MAML3* and *SETD7* are not the causal variants of the 93-Mb QTL. Conversely, for the 119-Mb region, the *ELOVL6:c.-533C > T* SNP was strongly associated with percentage of palmitic and palmitoleic FA, ACL, and C18:0/C16:0 and C18:1(n-7)/C16:1(n-7) elongation ratios. These results suggest pleiotropic effects of *ELOVL6:c.-533C > T* on BF and IMF FA composition.

## Competing interests

The authors declare that they have no competing interests.

## Authors’ contributions

JMF, MB and YRC conceived and designed the experiment. JMF was the principal investigator of the project. NI, MM and JMF collected samples. APO, JC, MB and MR performed the DNA and RNA isolation. MR, AC, JC and MB identified the polymorphisms and performed the genotyping. MR, YRC and JC performed the association analysis. MR, AC, APO and MB performed the gene-expression analysis. MR and JMF wrote the manuscript. All authors read and approved the final manuscript.

## Supplementary Material

Additional file 1: Table S1 List of SNPs genotyped. List of 144 SNPs located on SSC8 genotyped and genotyping statistics. Click here for file

Additional file 2: Table S2 Primers for *SETD7* and *MAML3* promoter sequencing (P), promoter and exon 1 sequencing (PE) and microsatellite genotyping (MS). **Table S3.** Significant SNPs affecting BF FA composition (FDR = 0.05) in LDLA analyses in the F2 generation. **Table S4.** Analysis of a two QTL model on SSC8 for the most significant regions affecting BF FA composition. **Table S5.** Additive value affecting BF FA composition in 168 F2 animals for the *SETD7:c.700G > T* and *ELOVL6:c.533C > T* SNPs. Click here for file

Additional file 3: Figure S1 Association study and LDLA of the C16:1(n-7), ACL and C18:1(n-7)/C16:1(n-7) elongation ratio in BF. Plot of association study (red points) and LDLA patterns (blue line) for palmitoleic acid, ACL and vaccenic/palmitoleic ratio; the X-axis represents positions in Mb on SSC8, and the Y-axis shows the –log10 (p-value); vertical, the pink line represents the position of the *MAML3* gene, the blue line represents the position of the *SETD7* gene and the green line represents the position of the *ELOVL6* gene on SSC8; horizontal, dashed ines mark the association study significance level (FDR-based q-value ≤ 0.05); positions in Mb are relative to the *Sscrofa10.2* assembly of the pig genome. Click here for file
