# Supplementary material for: New insight into the SSC8 genetic determination of fatty acid composition in pigs
Source: Genet Sel Evol. 2014 Apr 23;46(1):28. doi: 10.1186/1297-9686-46-28 (PMC4043687; doi:10.1186/1297-9686-46-28)
Supplement: Additional file 2: Table S2 — Primers for SETD7 and MAML3 promoter sequencing (P), promoter and exon 1 sequencing (PE) and microsatellite genotyping (MS). Table S3. Significant SNPs affecting BF FA composition (FDR = 0.05) in LDLA analyses in the F2 generation. Table S4. Analysis of a two QTL model on SSC8 for the most significant regions affecting BF FA composition. Table S5. Additive value affecting BF FA composition in 168 F2 animals for the SETD7:c.700G > T and ELOVL6:c.533C > T SNPs. [file 1297-9686-46-28-S2.docx]

**Table S2- Primers for SETD7** and **MAML3 promoter sequencing (P), promoter and exon 1 sequencing (PE), microsatellite study (MS) and** RT-qPCR (RT) study

| **Gene^1^** | **Primer** | **Sequence** | **Amplicon length (bp)** | **Tm** | **[MgCl_2_]** |
| --- | --- | --- | --- | --- | --- |
| *SETD7* | SETD7-Fw1 (P) | 5'-ACAACTTTCTCTTGCTCCCTTCTA-3' | 473 | 62ºC | 1.5mM |
| *SETD7* | SETD7-Rv1 (P) | 5'-ATTCAGAAATTCACCAGATCCAAA-3' |  |  |  |
| *SETD7* | SETD7-Fw2 (PE) | 5'-GTTCCTTTTCCGTTACCACAAC-3' | 478 | 62ºC | 1.5mM |
| *SETD7* | SETD7-Rv2 (PE) | 5'-GCTCAGAACTCCCGACCTC-3' |  |  |  |
| *SETD7* | SETD7-Fw (RT) | 5'-TGCTGGATATACTACCCAGATGGA-3' | 71 | 60ºC | np |
| *SETD7* | SETD7-Rv (RT) | 5'-TCTCCTGTCATCTCCCCATCTT-3' |  |  |  |
| *MAML3* | MAML3-Fw1 (P) | 5'-GTACCGCGCATTAAATAATATTCC-3' | 517 | 56ºC | 2.5mM |
| *MAML3* | MAML3-Rv1 (P) | 5'-GCCAGAAAACAGAGAAAGAAAGAT-3' |  |  |  |
| *MAML3* | MAML3-Fw2 (PE) | 5'-TGTATAACAACAACTTGGGCTCTC-3' | 663 | 58ºC | *GC-Rich PCR System |
| *MAML3* | MAML3-Rv2 (PE) | 5'-GACTGCAAAAGTAGATCGGTGA -3' |  |  |  |
| *MAML3* | MAML3_HEX-Fw (MS) | 5'- TGTATAACAACAACTTGGGCTCTC-3' | 249 | 58ºC | 2.5mM |
| *MAML3* | MAML3-Rv1 (MS) | 5'-GCCAGAAAACAGAGAAAGAAAGAT-3' |  |  |  |
| *MAML3* | MAML3_FAM-Fw (MS) | 5'-GCTGCCGTGTTTACTGAGCT-3' | 135 | 58ºC | 2.5mM |
| *MAML3* | MAML3-Rv3 (MS) | 5'-ACCATCACAATGATCAACTGCT-3' |  |  |  |
| *MAML3* | MAML3-Fw (RT) | 5'-GGTCAACCAGTTTCAAGGGTCT-3' | 102 | 60ºC | np |
| *MAML3* | MAML3-Rv (RT) | 5'-CCTGCATTCTGTGCCATCAA-3' |  |  |  |
| *ACTB* | ACTB-Fw (RT) | 5'-CAAGGACCTCTACGCCAACAC-3' | 130 | 60ºC | np |
| *ACTB* | ACTB-Rv (RT) | 5'-TGGAGGCGCGATGATCTT-3' |  |  |  |
| *B2M* | B2M-Fw (RT) | 5'-ACCTTCTGGTCCACACTGAGTTC-3' | 108 | 60ºC | np |
| *B2M* | B2M-Rv (RT) | 5'-GGTCTCGATCCCACTTAACTATCTTG-3' |  |  |  |
| *HPRT1* | HPRT1-Fw (RT) | 5'-TCATTATGCCGAGGATTTGGA-3' | 91 | 60ºC | np |
| *HPRT1* | HPRT1-Rv (RT) | 5'-CTCTTTCATCACATCTCGAGCAA-3' |  |  |  |
| *TBP* | TBP-Fw (RT) | 5'-CAGAATGATCAAACCGAGAATTGT-3' | 80 | 60ºC | np |
| *TBP* | TBP-Rv (RT) | 5'-CTGCTCTGACTTTAGCACCTGTTAA-3' |  |  |  |

^1^ The genes analyzed were: SET domain containing lysine methyltransferase 7 (*SETD7*) and mastermind-like 3 (*MAML3*).

np: not provided by the manufacturer.

**Table S3-** Significant SNPs affecting BF FA composition (FDR=0.05) in LDLA analyses in the F_2_ generation

| **Trait** | **Chromosomal region (Mb)** | **SNP** | **LR** | ***P*-value** | ***a* (SE)** |
| --- | --- | --- | --- | --- | --- |
| **C16:1(n-7)** | 93.87 | H3GA0025162 | 35.9228 | 2.05E-09 | 0.164 (0.085) |
|  | 119.85^1^ | INRA0030422 | 45.4291 | 1.58E-11 | 0.223 (0.082) |
| **ACL** | 93.72 | ALGA0048594 | 40.5341 | 1.93E-10 | -0.020 ( 0.001) |
|  | 117.66 | ALGA0049139 | 45.2997 | 1.69E-11 | -0.021 (0.001) |
| **C18:0/C16:0** | 93.87 | H3GA0025162 | 39.8781 | 2.70E-10 | -0.022 (0.002) |
|  | 119.73 | SIRI0000509 | 55.9362 | 7.48E-14 | -0.032 (0.002) |
| **C18:1(n-7)/C16:1(n-7)** | 93.87 | H3GA0025162 | 35.4047 | 2.68E-09 | -0.058 (0.016) |
|  | 117.66 | ALGA0049139 | 46.6404 | 8.53E-12 | -0.088 (0.015) |

LR. Likehood ratio test values; a (SE): additive effect (standard error).

^1^ SNP H3GA0025321 (119.89 Mb) showed the same *P*-value.

**Table S4-** Analysis of a two-QTLs model in SSC8 for the most significant regions
affecting BF FA composition

| **Trait** | **Chromosomal region (Mb)** | **SNP** | **LR** | ***P*-value** |
| --- | --- | --- | --- | --- |
| **C14:0** | 91.57 | ALGA0048513 | 21.0699 | 4.43E-06 |
|  | 117.66 | ALGA0049139 |  |  |
| **C16:0** | 91.57 | ALGA0048513 | 47.4716 | 5.58E-12 |
|  | 117.66 | ALGA0049139 |  |  |
| **C18:0** | 91.56 | H3GA0025111 | 19.1441 | 1.21E-05 |
|  | 119.73 | SIRI0000509 |  |  |
| **C16:1(n-7)** | 91.56 | H3GA0025111 | 68.9761 | 1.11E-16 |
|  | 119.73 | SIRI0000509 |  |  |
| **C18:1(n-9)** | 91.57 | ALGA0048513 | 35.9079 | 2.07E-09 |
|  | 117.66 | ALGA0049139 |  |  |
| **C20:2(n-6)** | 91.56 | H3GA0025111 | 23.3687 | 1.34E-06 |
|  | 117.55 | ALGA0049135 |  |  |
| **ACL** | 91.57 | ALGA0048513 | 69.2893 | 1.11E-16 |
|  | 117.66 | ALGA0049139 |  |  |
| **C16:1(n-7)/C16:0** | 91.57 | H3GA0025111 | 37.5734 | 8.80E-10 |
|  | 119.73 | SIRI0000509 |  |  |
| **C18:0/C16:0** | 91.56 | H3GA0025111 | 66.7598 | 3.33E-16 |
|  | 119.73 | SIRI0000509 |  |  |
| **C18:1(n-7)/C16:1(n-7)** | 91.57 | ALGA0048513 | 66.3888 | 3.33E-16 |
|  | 119.73 | SIRI0000509 |  |  |

LR. Likehood ratio test values.

**Table S5-** Additive value affecting BF FA composition in 168 F_2_ animals for *SETD7:c.700G>T* and *ELOVL6:c.533C>T* SNPs

| **Trait** | ***SETD7:c.700G>T*** | ***ELOVL6:c.533C>T*** |
| --- | --- | --- |
| **C16:0** | 0.421 | 0.652 |
| **C16:1(n-7)** | 0.169 | 0.221 |
| **C18:1(n-9)** | -0.465 | -0.714 |
| **C18:1(n-7)** | 0.068 | 0.081 |
| **ACL** | -0.015 | -0.022 |
| **MUFA** | -0.266 | -0.457 |
| **C16:1(n-7)/C16:0** | 0.006 | 0.007 |
| **C18:0/C16:0** | -0.022 | -0.032 |
| **C18:1(n-7)/C16:1(n-7)** | -0.062 | -0.101 |
| **C20:2(n-6)/C18:2(n-6)** | -0.003 | -0.005 |
