# Supplementary figures and images for: New insight into the SSC8 genetic determination of fatty acid composition in pigs
Source: Genet Sel Evol. 2014 Apr 23;46(1):28. doi: 10.1186/1297-9686-46-28 (PMC4043687; doi:10.1186/1297-9686-46-28)

**C16:1(n-7)**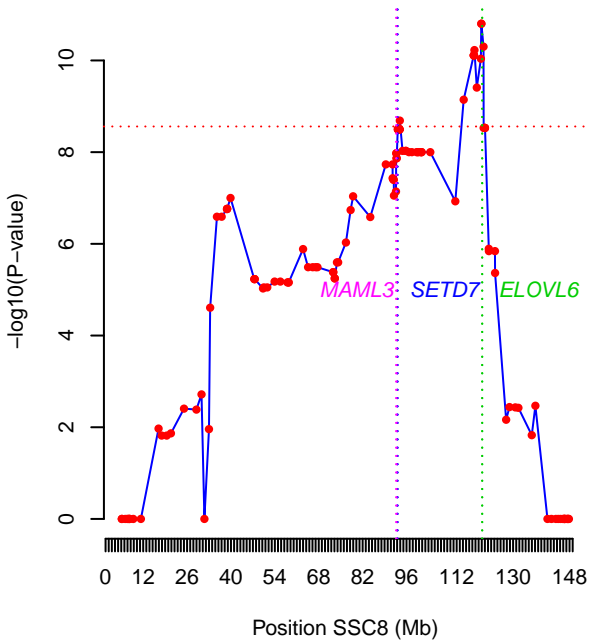**ACL**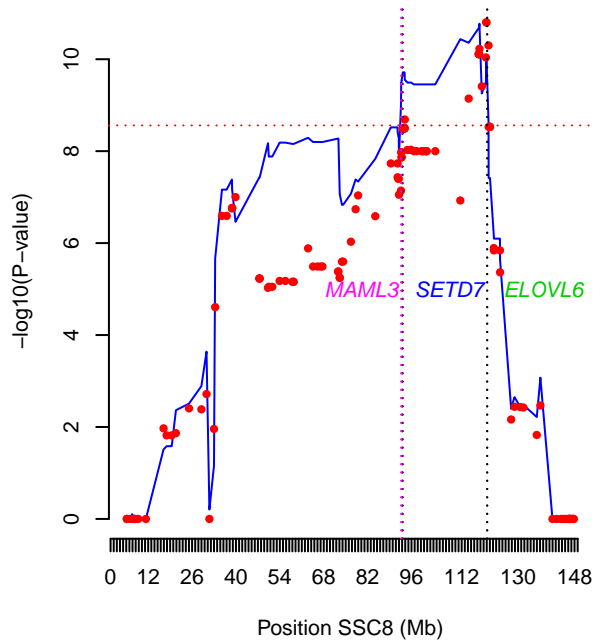**C18:1(n-7)/C16:1(n-7)**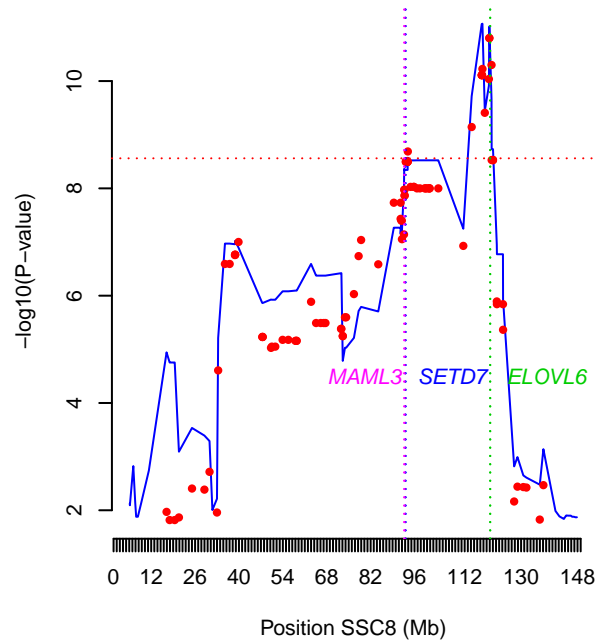

Supplement: Additional file 3: Figure S1 — Association study and LDLA of the C16:1(n-7), ACL and C18:1(n-7)/C16:1(n-7) elongation ratio in BF. Plot of association study (red points) and LDLA patterns (blue line) for palmitoleic acid, ACL and vaccenic/palmitoleic ratio; the X-axis represents positions in Mb on SSC8, and the Y-axis shows the –log10 (p-value); vertical, the pink line represents the position of the MAML3 gene, the blue line represents the position of the SETD7 gene and the green line represents the position of the ELOVL6 gene on SSC8; horizontal, dashed ines mark the association study significance level (FDR-based q-value ≤ 0.05); positions in Mb are relative to the Sscrofa10.2 assembly of the pig genome. [file 1297-9686-46-28-S3.pdf]
